# The role of principled engagement in public health policymaking: the case of Zambia’s prolonged efforts to develop a comprehensive tobacco control policy

**DOI:** 10.1080/16549716.2023.2212959

**Published:** 2023-05-22

**Authors:** Adam Silumbwe, Miguel San Sebastian, Joseph Mumba Zulu, Charles Michelo, Klara Johansson

**Affiliations:** a Department of Health Policy and Management, School of Public Health, University of Zambia, Lusaka, Zambia; b Department of Epidemiology and Global Health, Umeå University, Umeå, Sweden; c Department of Epidemiology and Biostatistics, School of Public Health, University of Zambia, Lusaka, Zambia; d Strategic Centre for Health Systems Metrics (SCHEME), Global Health Institute, Nkwazi Research University, Lusaka, Zambia

**Keywords:** Collaborative governance, principled engagement, tobacco control, policy process, Zambia

## Abstract

**Background:**

The Framework Convention on Tobacco Control (FCTC) requires countries to develop and implement multi-sectoral tobacco control strategies, including policies and legislation. Zambia, potentially faced by a rising problem of tobacco smoking, signed the FCTC in 2008 but has been unable to enact a tobacco policy for over a decade.

**Objective:**

This study explores the role of ‘principled engagement’, a key element of the theoretical framework for collaborative governance, in Zambia’s delayed success to develop a comprehensive tobacco control policy.

**Methods:**

This was a qualitative case study of key stakeholders in the collaborative process of trying to develop a tobacco policy in Zambia. Participan-ts were sampled from across various sectors, including government departments and civil society, comprising anti-tobacco activists and researchers. A total of 27 key informant interviews were undertaken. We supplemented the interview data with a document review of relevant policies and legislation. Data were analysed using thematic analysis.

**Results:**

Several factors hindered efforts to attain principled engagement, including the adverse legal and socioeconomic environment in which the collaborative regime evolves; poor planning of meetings and frequent changes in tobacco focal point persons; lack of active and meaningful participation; and communication challenges among the key stakeholders. These collaborative dynamics, coupled with the opposition to tobacco control efforts from within some government departments, revealed the inadequacy of the current collaborative governance regime to facilitate enactment of a comprehensive tobacco control policy in Zambia.

**Conclusion:**

Efforts to develop a comprehensive tobacco control policy in Zambia will require addressing challenges such as disagreements, communication, and leadership at engagement level across interested sectors. We further argue that principled engagement has a greater role to play in unlocking these efforts and should therefore be embraced by those entrusted to lead the process to develop tobacco policy in Zambia.

## Background

### Global policy and legal framework to address tobacco smoking and implementation challenges

Tobacco smoking is a leading cause of preventable death, and reducing its use is central to global health policy initiatives [1]. Because tobacco policy development has been shown to be such a fraught process in many countries, it is a relevant example of potential challenges in health policy making [2]. Target 3.4a of the sustainable development goals calls for strengthening implementation of the Framework Convention on Tobacco Control (FCTC), a global health treaty that establishes evidence-based standards to address demand and supply factors driving tobacco use [3,4]. As of 2020, the FCTC has been ratified by 180 of the 194 World Health Organisation (WHO) member countries [5]. The FCTC requires countries to develop and implement tobacco control strategies including policies and legislation that protect people from second-hand smoke, restrict advertising and prohibit tobacco sale to minors [6]. However, the FCTC recommendations fail to consider the innate complexity and contestation of health policy development and implementation within country contexts.

Similar to most health policies, implementation of the FCTC, particularly in LMICs, has faced numerous challenges, with most countries unable to fully achieve the commitments made in the treaty [5,7,8]. Programmes to reduce tobacco consumption remain unremittingly underfunded. Only 2% of global development funding goes towards tobacco control, according to the WHO [1]. Another challenge is the interference of the tobacco industry, which stifles programmes, legislation and policies seeking to reduce tobacco production and use [9–11]. Key interference tactics include corporate social responsibility activities, lobbying, litigation, alleged bribery and arguments on illicit tobacco trade [12].

### African countries’ progress towards adoption of the FCTC policy and legal measures

Despite the challenges, some African countries in the WHO region have made progress in implementing some of the FCTC commitments across various health policies and laws. In 2015, 32 of 43 African countries had laws restricting tobacco advertising, promotion and sponsorship [6]. African countries have continued to strengthen their tobacco control mechanisms, including developing tobacco control programmes and plans of action [13]. These measures have largely been driven by the health sector, yet the FCTC recommends use of multi-sectoral structures to accelerate development and implementation of comprehensive tobacco demand and supply control strategies [14–16]. Multisector engagement has been shown to be a challenge in other health policy making processes across the world [17].

### The institutional context of Zambia tobacco control efforts

Zambia is one of the African countries facing an imminent increase in tobacco use that have ratified the FCTC but are yet to develop a comprehensive national policy response addressing both tobacco demand and supply[18]. Efforts to come up with a tobacco policy have been delayed for over a decade now, and when it is likely to be enacted is unknown [19]. The country’s weak economy, strong tobacco industry presence, as well as poor coordination and collaboration among institutional stakeholders, have been reported as key factors impeding the development of a tobacco policy [20–22]. On the other hand, Zambia has statutory instruments, such as the 163 of 1992 and 185 of 2008, concerned with regulating labelling, prohibition of commercial advertising, sale of tobacco to children, and prohibition of smoking in public places, respectively [23,24]. However, these are only examples of subordinate legislation focusing on consumption and have not had the desired impact on tobacco use. A comprehensive tobacco policy is the main goal of tobacco control efforts, but to date this remains elusive in Zambia.

### The tobacco policy process in Zambia

After ratifying the FCTC in 2008, Zambia initiated the process of developing a comprehensive tobacco policy in the following year. This was accompanied by the assignment of tobacco focal point persons in all government departments and related sectors, who are responsible for coordinating tobacco activities in their respective sectors. Multiple government sectors and key non-governmental stakeholders have been engaged in consultative meetings to discuss the development and content of a comprehensive tobacco policy since 2009. The main stakeholders in these meetings from the government side have included the Ministries of Health, Agriculture, Trade and Commerce, Local Government and Housing, Justice, and Community Development and Social Welfare. Some civil-society organisations such as the Centre for Policy and Trade, WHO, the Non-communicable Diseases Alliance, and anti-tobacco and alcohol organisations have also been involved. The meetings were planned to be held every four months each year, with the Ministry of Health as the main convenor and the WHO country office providing resources and technical assistance. To date, the meetings have yielded two draft policy documents that the cabinet has been unable to approve. The cabinet, which is a body of government ministers that approves legislation to parliament, has on both instances requested further consultations, because according to them, the draft policy content lacked the representation and consensus of all tobacco stakeholders [25].

Whilst there are studies that have looked at the institutional context of tobacco production and control in Zambia [19–22], no studies have explored the collaborative engagement process among institutional stakeholders and how it has influenced the development of a tobacco control policy in Zambia. Most studies on the contested tobacco policy process in Zambia have paid little attention to engagement within the collaborative governance regime and how it has affected policymaking. Understanding the reasons for the success or failure of the engagement processes among these stakeholders within the collaborative spaces for tobacco control may be important in shaping future advances in not only efforts to develop tobacco policy but also general health policies in Zambia. As part of a broader project assessing intersectoral collaborative governance regarding tobacco policy in Zambia, this study sought to explore the role of ‘principled engagement’, a key element of the theoretical framework for collaborative governance, in Zambia’s delayed success to develop a comprehensive tobacco control policy. We focus on principled engagement because it is considered the initial activity in any collaborative governance process of any policy making undertaking.

## Methods

### Integrative framework for collaborative governance

Collaborative governance is the process of public policy decision-making and management that engages people constructively across sectors to carry out a public purpose. We used Emerson’s integrative framework for collaborative governance to explore the collaborative dynamics among stakeholders involved in the development of the tobacco policy in Zambia [26]. The theoretical framework categorises several multilevel factors of the internal dynamics and causal pathways of the collaborative governance process and its performance. It comprises three nested dimensions, representing the general systems context, the collaborative governance regime, and its collaborative dynamics and actions, which over time shape the extent to which such a regime is effective. Collaborative dynamics involve the interactive components of principled engagement, shared motivations, and capacity for joint action.

**Figure 1.**
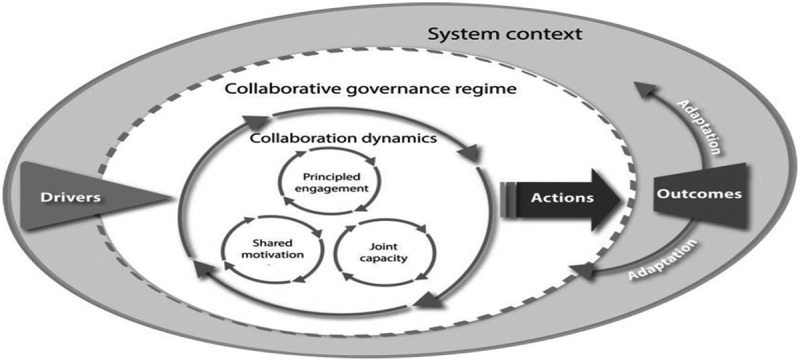
Emerson’s integrative framework for collaborative governance [19].

In this study, the consultative meetings to develop a comprehensive tobacco policy in Zambia constitute our collaborative governance regime of interest. We focused on principled engagement as a first step for understanding the collaborative dynamics within these meetings. We posited that principled engagement would be a vital activity setting in motion the rolling wheels of collaboration and would be largely responsible for the success and failure of the dynamics in any collaborative undertaking (Figure 1). Through principled engagement, people with differing goals regarding content, relations and identity work across their respective sectors to create common value [27,28]. We adapted Emerson’s framework to explore stakeholders’ participation, representation of key sector interests, communication among sectors and openness and flexibility in discussing opposing views during the meetings as key elements of the principled engagement process. To do this, we also describe the legal and socioeconomic context surrounding efforts to develop a tobacco policy to better appreciate how principled engagement is shaped by the external environment.

## Study design

This was a qualitative case study design using two methods of data collection: document review and informant interviews with key stakeholders. The case study comprised an in-depth exploration of the functions and operations of the key institutional stakeholders in the consultative meetings to develop a tobacco policy in Zambia.

### Document review

We conducted a document review to understand the broader systemic context of institutional stakeholders in tobacco control in Zambia. These documents included all those governing the supply and demand side sectors, including acts and laws, strategic plans, and guidelines. Some documents were specific to tobacco production and marketing, while others governed tobacco consumption and use. Some documents had a national development focus, while others addressed non-communicable diseases. Further, we reviewed policy documents specific to the health sector that explicitly recognised tobacco as a critical risk factor for certain health conditions. We sought to understand the stance of these policy documents on tobacco consumption control and how supportive or not the policy and legal environment is to engagement in the collaborative governance regime. Data from the document review were used to triangulate the information from the key informant interviews. We extracted the data from the legal and policy documents using a predefined data grid on key aspects, such as the year of the law or policy, the objectives/focus, and its position on collaboration (Tables A1 & A2). These documents date from 1930 to 2021 [23,24,29–31]

## Study population

The study population consisted of stakeholders that have been attending the consultative meetings to come up with a tobacco policy since the commencement of the process in 2009. The participants for this study came from a variety of public and civil society sectors of the economy. Specifically, the civil society comprised of organisations whose main activities are tobacco use control, primary care research, commerce and trade, as well as the local network of organisations against NCDs in Zambia (Table 1).

**Table 1.** Study participants.

Sector	Number of Interviews
Civil Society	4
Ministry of Local Government	2
Ministry of Labour	1
Ministry of Health	5
Ministry of Agriculture	4
Ministry of Commerce	2
Ministry of Education	2
Ministry of Finance	3
World Health Organisation	2
Ministry of Justice	2
**Total Interviews**	**27**

## Sampling and recruitment of study participants

We used both purposive and snowball sampling to recruit the study participants. Through the Ministry of Health, as well as checking attendance registers and meeting minutes, we were able to gather information on the tobacco focal point persons across sectors who have been attending the consultative meetings. We were also able to contact those who had participated earlier in the process – former focal persons – which allowed us to gain an understanding of how these collaborative meetings had evolved over time.

## Data collection tools and techniques

We conducted key informant interviews with a total of 27 (*n* = 27) stakeholders to unpack the collaborative dynamics within the consultative meetings to develop a tobacco policy (Table 1). The total number of interviews was reached using the principle of theoretical saturation, which stipulates that one stops conducting interviews when the additional data do not provide new information [32]. We used the study framework to inform the development of the interview guide and framing of the questions. The interview guide (Appendix B) encompassed questions on how the broader political, legal, and social economic context – including tobacco-related policies and legislation, and institutional practices – shaped collaborative engagement efforts. We asked the participants to mention and explain how specific Zambian tobacco policies and laws affected the collaborative dynamics in tobacco meetings. Further, the guide also explored issues related to stakeholders’ participation, representation of key sector interests and communication among the sectors as well as openness and flexibility in discussing opposing perspectives. For example, we asked the participants to describe the participation of various sectors during the meetings, what they thought about representation of key tobacco stakeholders as well as how they perceived the communication during and after the meetings. Each of the interviews lasted between 35 minutes and 1 hour and were held at a location convenient to the respondents. For those who were unable to meet physically, some interviews were also conducted using the online meeting platform Zoom. All interviews were conducted in English, audio recorded and transcribed verbatim.

## Data analysis

We used thematic analysis to analyse the data [33]. Thematic analysis facilitates identification and making sense of common themes in participants discussion of the research topic. NVivo (version 12 Pro, QSR International) software was used to facilitate the data management, coding, and analysis. Coding was done by the first author, in constant discussion with all co-authors. Firstly, the first author familiarised himself with the data through field note reports and reading the verbatim transcriptions. Second, initial codes were identified by the first author who carefully read through the first five transcripts and manually labelled portions of text. Initial codes were discussed in the full author group, then applied to the whole material. A coding structure comprising thematic definitions and meaning was developed and imported in NVivo 12 Pro for coding of the entire dataset. Once transcripts were coded, the third phase comprised construction of themes – patterns and relationships within the coded data. This involved reviewing the initial codes, collapsing, realigning, and clustering them into major and sub-themes. The data analysis approach was twofold; deductive in that the main themes under the general systems context affecting collaborative engagement were informed by concepts in the study framework; and inductive in that some of the main themes under the thematic areas relating to principled engagement emerged from the data (Table 2).

**Table 2.** Major themes and sub-themes of the general systems context and factors shaping principled engagement in the tobacco policy process.

Major theme	Sub-themes
**General systems context affecting collaborative engagement**	
Unfavourable legal context	Conflicting intra-sector tobacco laws and policies Lack of enforcement of the tobacco-free laws
Adverse socioeconomic context	Tobacco contributes to national economy Tobacco industry interference in some sectors
**Thematic areas relating to the elements of principled engagement**	
Poor planning of meetings and frequent changes in tobacco focal point persons	Irregular meetings and prolonged consultation Frequent changes in tobacco focal point persons
Lack of active and meaningful participation	Local government feel they are assigned a passive role Some sectors take less leadership on tobacco control within their organisations Sectors assigning junior staff to attend meetings
Communication challenges among the key stakeholders	Non-functional communication channels Limited communication among sectors outside the consultative meetings Communication hampered by the view that public health conflicts with social-sector goals Flexibility and open mindedness vital to enhancing communication Need to build appropriate communication mechanisms

## Ethical considerations

Ethical approval was sought from the Excellence in Research Ethics and Science (ERES) Converge ethics committee as well as the Zambia National Health Research Authority (ZNHRA) (ref. no. 2019-Dec-007). During data collection, participants were provided with relevant information about the study, ensuring clarity about possible benefits and risks, so they could make informed decisions about whether or not to participate, and consent was sought from them. Most of the interviews were carried out at participants’ workplace, so no transport refunds were provided. All study participants were de-identified by providing them with participant codes during both data collection and analysis to ensure confidentiality. Data collected were treated with utmost privacy and securely stored on a password-protected hard drive. This research was performed in accordance with the Declaration of Helsinki [34].

## Results

Several thematic areas emerged from the qualitative data as shaping the principled engagement in the collaborative meetings to develop a tobacco policy in Zambia. We present the results in two parts. First, we present the thematic areas of the general system context in which tobacco policy process has developed: the unfavourable legal and the adverse socioeconomic context. Second, we describe key themes related to the core process elements of principled engagement: poor planning and frequent changes in tobacco focal point persons, lack of meaningful participation and imbalanced representation of diverse sector interests and limited communication among the key stakeholders (Table 2).

## General system context affecting collaborative engagement

### Unfavourable legal context

The stakeholders revealed that there were various pieces of legislation that contradicted efforts to address tobacco demand and supply in Zambia; for example, the Tobacco Act, the Zambia Development Agency Act, and the 7th National Development Plan all promote tobacco production, including providing incentives for tobacco value addition such as cigarette manufacturing. The Ministry of Agriculture argued that there was no need for a new tobacco policy, but that the government should rather strengthen the existing laws, such as the Public Health Act, to address consumption. On the other side, the Ministry of Health and Civil Society argued that the existing policies contradicted the FCTC recommendations of scaling down tobacco production, hence the need for a comprehensive policy. As one civil society stakeholder stated:

The Zambia Development Agency Act is very clear when it comes to investments including tobacco. When investors come into the country, it incentivizes or encourages them to bring more investments. So, that conflicts with the FCTC recommendations that you are not supposed to incentivize the tobacco industry. The policies provide tax rebates, where investors go one year without subjecting them to any form of taxation. (R019, Civil Society Official)

In Zambia, only two subsidiary laws address tobacco consumption, the statutory instruments 163 of 1992 and 185 of 2008 [23,24]. According to the Ministry of Health, the Ministry of Local Government and civil society, the government has not actively enforced either law since they were enacted. They explained that the lack of clarity in existing laws makes their enforcement in public places challenging. Further, the inadequate security personnel in public places, such as markets, as well as poor funding of the Ministry of Local Government, has also made it difficult to implement these laws. A local government official explained:

For example, if I establish a bar or a restaurant, the statutory instrument says you should have a smoking zone without really defining what a smoking zone is. So, you get to a restaurant, someone is by the corner, they say this is our smoking zone. What can you tell them? It is not defined in the law what the smoking zone should be. So, you will be unable to punish or convict if you take the matter to court. (R024, Local Government Official)

### Adverse socioeconomic context

Stakeholders from sectors, such as the Ministries of Finance, Commerce and Agriculture indicated that the poor state of the Zambian economy reflected in the high levels of unemployment and national debt, as well as the contracting economy, has made it difficult for the government to avoid revenue from the tobacco industry. A Ministry of Finance official indicated:

So, the treasury needs money, all the little money plays an important role in financing development programmes … . So, when you remove the contribution of the tobacco industry that it is something very significant on the budget. (R009, Ministry of Finance Official)

On the other hand, the Ministry of Health and civil society had a different perspective on the economic argument. They argued that the tobacco industry has been inflating the figures of the number of people it employs to show that that it affects many lives, that farmers should instead be supported to grow alternative cash crops and that the revenues collected from the tobacco industry were serving to offset health care costs. One civil society official commented:

There’s a lot of deception from the tobacco industry which has locked our people in a cycle of poverty. As it is now, no small-scale tobacco farmers make a profit out of tobacco growing. The Ministry of Agriculture is supposed to support the draft tobacco bill because it recommends that the Ministry of Agriculture should be encouraging its tobacco farmers to change to alternative crops that will make them profit. (R001, Civil Society Official)

Both the Ministry of Health and civil society stakeholders reported that the tobacco industry was interested in suppressing the enactment of a tobacco policy, because regulating consumption would limit their market. Mentioned examples of interference strategies by the tobacco industry included meeting political players, lobbying legislators, and presenting counterevidence on tobacco control to senior government officials. A civil society official reflected:

From my experience it’s the interference of tobacco industry that has caused the delay, there I cannot deceive you; the tobacco industry is very interested to see that this law doesn’t exist in Zambia. They are looking at the profit margin for their companies, but we were looking at the public health, at the health of a human being who’s being destroyed by the tobacco use. (R006, Civil Society Official)

On the contrary, representatives from the Ministries of Finance and Agriculture rejected the notion of interference by the tobacco companies in their policymaking. They considered involving the tobacco industry in discussions related to the economy of the country, such as tobacco taxes, as part of their mission. An official from the Ministry of Finance observed:

So, if we have someone from the Ministry of Finance, for example, maybe who works for budget office and deals with taxes. By the virtue of them dealing with taxes, it means that you cannot forbid their interaction with the tobacco industry. The tobacco industry will always want to consult with us where they have tax concerns. That does not mean we are working with the industry against Ministry of Health, no! (R009, Ministry of Finance Official)

## Thematic areas relating to the elements of principled engagement

### Poor planning of meetings and frequent changes in tobacco focal point persons

All of the stakeholders described the consultative meetings, organised by the Ministry of Health, as multisectoral in nature. However, they felt that the consultations to develop a tobacco policy have gone on for too long without yielding a policy document. Stakeholders from outside the health sector noted that the consultative meetings were more irregular than originally planned, but more frequent around international events, such as the conference of parties by the FCTC secretariat. An official from the Ministry of Commerce narrated:

The past two years I haven’t seen much activity in terms of multisectoral activity like we had seen before, especially running up to the Geneva convention. Cause after we came back from there really, there haven’t been much coming together to look at what is happening to the tobacco policy itself, what is happening to the tobacco policy. There hasn’t been much to hear from all the stakeholders. Personally, I haven’t been consulted. (R005, Ministry of Commerce Official)

More so, the Ministries of Finance, Commerce and Local Government noted that on some occasions invitations/notifications to the consultative meetings were shared late, sometimes a few hours or a day before. This, they said, affected their preparations and participation in the meetings. As one official from the Ministry of Finance commented:

There are certain times I used to have experiences where you get a phone call that there is a meeting that is supposed to take place, but you have not seen the invitation letter. But I need clearance from my senior to attend the meeting. I think that there is a need to ensure that the invitations are sent as quickly as possible so that people are prepared, and people have enough time to prepare what they are going to discuss and try to think through the submissions to make. I think it would be very easy like that. (R016, Ministry of Finance)

The frequent changes in sector representatives in the meetings due to a continuous restructuring in the ministries were also said to affect the engagement process. Sometimes the tobacco focal persons were transferred to other departments or another ministry entirely. This meant reassigning a new person who would have to be retrained on tobacco issues:

The challenge comes when you have a meeting, a different set of people come, whereas those that attended the other meeting are not seen, hence drawing the progress backwards. So, before the meeting you must start explaining to the participants over and over and that’s where you might start having back and forth. (R009, Ministry of Health Official)

### Lack of active and meaningful participation

All of the stakeholders indicated that the consultative meetings included most of the key tobacco stakeholders, although there was inadequate space for participation in the discussions. For example, stakeholders from the Ministry of Local Government argued that they were mostly assigned a passive role, even when they were the main implementers of a smoke-free policy in public places as one local government officer described:

There was a point where, during the early stages of policy formulation, the local government was left out. I remember I had a chat with someone from the organizing committee. I had to say now you’re leaving out the people who enforce. Where are you going to get the insights on enforcement? Oh, you can come up with the law, a law which is not enforced, then it’s useless. (R024, Ministry of Local Government Official)

The Ministry of Health representatives mentioned that certain participants attended the consultative meetings because they had been ordered to and not because they saw themselves as tobacco control leaders in their respective sectors. Further, they indicated that one of the major challenges has been that government departments assign junior representatives whose submissions are later overruled by the higher authority in their respective sectors.

### Communication challenges among the key stakeholders

All of the stakeholders indicated that the communication channels among key sectors on issues of tobacco control were not functioning as expected. For example, the Ministry of Health complained that there was a failure to communicate what was discussed in the consultative meetings to the respective sectors by the tobacco focal point persons. Further, the stakeholders from the Ministries of Finance and Commerce stated that there was little communication on matters of tobacco control among the sectors between the consultative meetings. Active communication on tobacco-related issues seemed only to take place towards or during the consultative meetings, as explained by a stakeholder from the Ministry of Finance:

What I have noticed is that the communication among the sectors with regards to tobacco control hasn’t been that effective. For instance, you only hear about certain issues when we are about to have a meeting. I think if we are to make progress in addressing the contentious issues in the proposed policy, we need more effective communication and sharing of information on tobacco. (R015, Ministry of Finance Official)

Communication among the stakeholders was hampered by the perception that the public health control of tobacco conflicted with other goals, such as livelihood and state finances. There were complaints about the lack of openness in discussing concrete plans for alternative livelihoods for tobacco farmers, as well as alternative uses of tobacco such as fertiliser. The agriculture and commerce sectors felt that being open minded and flexible would help to enhance communication and understanding among the stakeholders.

I think the Ministry of Health should go into these meetings with an open mind to be able to learn and influence stakeholders from other government departments, to understand why it’s important for the Zambian people and its economy to regulate tobacco consumption. (R020 Ministry of Agriculture Official)

Some civil society stakeholders suggested that there was a need to network more and build appropriate communication mechanisms for a coordinated approach to influence the tobacco policy as one of them described:

There is a need for communication systems to be in place for coordination of the multisectoral activities on tobacco control in Zambia. So, in every organization you need to have systems so that tobacco information flows within and among the sectors. If we are to succeed in the development of the policy, we need all sectors to have the information they need on tobacco. (R002, Civil Society Official)

## Discussion

This study explored the role of ‘principled engagement’, a key element of the theoretical framework for collaborative governance, in Zambia’s delayed success to develop a comprehensive tobacco control policy. Several factors were identified as hindering the efforts to attain that engagement, including the adverse legal and socioeconomic environment in which the collaborative regime is evolving; poor planning of meetings and frequent changes in tobacco focal point persons; lack of active and meaningful participation; and communication challenges among the key stakeholders. These collaborative dynamics, coupled with certain opposition from within government departments, revealed the inadequacy of the current collaborative governance regime to facilitate the enactment of a comprehensive tobacco policy in the country.

## Adverse legal and socioeconomic context

A supportive legal and socioeconomic environment is vital for meaningful engagement in any policy development process. However, we found that there were various sector-specific policies that inhibited the tobacco policy process [29,31]. For example, the economic policy of providing incentives to expand tobacco manufacturing contradicted the FCTC recommendations of scaling down production, as reported elsewhere [20,21,35]. In addition, we found that some sectors did not see the need for a new tobacco policy, arguing that the government should rather focus its resources on strengthening the existing tobacco policies. The existing policies are, however, not only inadequate, but also never entirely enforced and lack supply side measures [20]. A new tobacco policy would help to harmonise action across sectors. For instance, the policy implementing a smoking ban in public places is only enforced in healthcare facilities but not in spaces, such as markets or bus stations, because they have no designated smoking areas and the enforcement capacity by the local authorities is lacking [19,20,36]. In the case of the Kenyan tobacco policy process, the inadequacies of their existing tobacco policies were seen by the various sectors as a major driver in the development of a comprehensive policy to harmonise and strengthen tobacco control efforts [37]. Unfortunately, this is not the path that Zambia has taken yet.

The tobacco industry’s influence in the policy process, including meeting political players, lobbying legislators and presenting counterevidence on tobacco control, has been a major impediment to the development of a comprehensive tobacco policy, as reported in similar studies [9,19]. The industry’s political and economic power, whilst taking advantage of the loopholes in the government’s efforts to grow the local economy, continues to place tobacco production and control in conflict [22]. Other studies – including the investment case for tobacco control in Zambia report of 2019 by the WHO – have, however, clearly disputed that the economic contribution of the tobacco industry is significant [20,38]. These studies have found that the economic contribution to the Zambian treasury is overstated and pales when compared to the long-term health effects of tobacco use and the burden it places on the national economy [20].

## Factors shaping the principled engagement

In addition to the challenging adverse legal and policy environment, the selection of junior representatives as tobacco focal point persons, as well as the frequent changes in sector representatives, has negatively affected principled engagement in the tobacco policy process. These issues may explain the weak commitment accorded to tobacco control activities by key decision-makers within the government set-up, which ultimately negatively affects the ownership and legitimacy of the tobacco policy process. A study comparing the tobacco policy process between Iran and Egypt highlighted how Iran had advanced tobacco control policies because of the political commitment shown by their government through the allocation of appropriate resources and personnel, while nothing progressed in Egypt due to the lack of such commitment [39].

Active and meaningful participation is a key element of the success of any policy process because it ensures multiple perspectives and interests. For example, in a South African study, it was reported that one of the key facilitators to the formulation of the tobacco policy was the diverse, proactive and dynamic nature of the stakeholders, which provided the social capital to enhance engagement and good-will [40]. However, we found that sectors such as the Ministry of Local Government felt left out and consigned to insignificant roles in the tobacco policy process, although they are the custodians of the only existing tobacco consumption policies in the country. Studies have shown that the tobacco policy process succeeds if multiple stakeholders, with different concerns and interests often in competition with one another, are actively involved [41,42].

Additionally, the poor communication found in this study probably contributed adversely to the policy process. According to Labonte et al., poor communication negatively affects the coordination and collaboration among institutional stakeholders, which leads to an environment that impedes the development of a comprehensive tobacco policy [20]. The absence of a whole-of-government approach towards tobacco control has probably resulted in the lack of a clear communication strategy – hence some government ministries working at cross-purposes. For instance, Namibia and Nigeria succeeded in passing tobacco policies through the use of a highly effective communication strategy that engaged traditional and social media [43,44]. Similarly, in South Africa and Togo, the tobacco policies formulated and implemented relied heavily on information transfer between stakeholders and less on a collaborative problem-solving approach [40].

## Strengths and limitations

The use of the integrative framework for collaborative governance facilitated an in-depth exploration of the collaborative dynamics in the tobacco policy process to explain how and why certain strategies are not working and may indeed be contributing to the delayed enactment of a comprehensive tobacco policy in Zambia. We triangulated information from the document review and the key informant interviews, as well as the views among the various sector stakeholders that participated, to ensure the trustworthiness and validity of our findings. Additionally, the iterative reading, reflections, and discussions on the collected and coded data by the entire research team allowed for constant comparisons, which also increased the validity of the emergent themes.

We acknowledge that our study has several limitations. First, the purposively selected sample of a few heads of government departments may not give a comprehensive picture of why the tobacco policy has been delayed, given that all authority and power to approve or reject policy lies with the Cabinet of Ministers. Second, the challenge of conducting interviews with policymakers who might not dedicate sufficient time or only provide limited answers to protect their positions may limit the depth of data collected. In addition, although efforts were made to interview many stakeholders, some of them were unavailable, despite several attempts to schedule interviews. Finally, having only one person to code the data may affect the validity of our findings. Despite these limitations, we believe that our findings contribute with valuable knowledge to understanding tobacco policy processes, not only in Zambia, but in other low- and middle-income countries.

## Conclusion

We found the presence of inherent challenges suggesting the inadequacies of the current collaborative governance regime to facilitate the enactment of a comprehensive tobacco policy in Zambia. Identified challenges included aligning the legal and socioeconomic environment to support tobacco supply side measures, enhancing policy development planning meetings, encouraging active and meaningful participation, and improving communication among stakeholders. These challenges pose a threat to shared outputs of consultative engagement in not only the tobacco policy process, but most health policy processes in low- and middle-income countries. Developing a comprehensive health policy in a context of diverse and conflicting sector interests is a delicate process that requires carefully structured strategies to strengthen principled engagement, which has the potential to unlock the required trust, commitment and leadership needed to enhance collaboration. We recommend that future research seeks to understand how addressing some of the impediments to principled engagement can move forward the process of health policy formulation in Zambia and other similar countries. Further, future research may also employ a stakeholder analysis to place the actors along the spectrum of influence and expertise, to better understand the principled engagement dynamics in the collaborative health policy making process. Future research could also compare and share lessons with similar health policy processes that have succeeded in other settings.

## Paper context

In Zambia, efforts to come up with a tobacco policy have been ongoing for over a decade and the country is not yet close to developing one. Few studies have explored the collaborative dynamics within the tobacco policy process and the potential role of principled engagement. This paper posits that principled engagement is a vital activity that sets in motion collaboration and is responsible for the success of any collaborative undertaking of the tobacco policy process.

## Appendix A

**Table A1.** Document review of tobacco laws and policies in Zambia.

Laws	Objective of the law	Position on collaboration in tobacco consumption control
1. Public Health Act, No. 12 of 1930 (as amended to Act No. 22 of 1995)	The Act provides for the notification, prevention and suppression of infectious diseases, regulates all matters concerned with public health including water, food supplies sanitation and housing.	Does not clearly state the role of collaboration in tobacco consumption control efforts, focuses more on infectious diseases control and general matters of public health
2. Tobacco Act No. 64 Chap. 237	The Act provides for the promotion, control and regulation of production, marketing and packing, control of export and import of tobacco, the direction and promotion of research in connection with tobacco.	Not concerned with tobacco consumption control, but rather the main focus is on marketing and production.
3. Tobacco Mktg & Licensing Rules – subsidiary of the tobacco Act	Provide rules for licensing of graders, buyers, auction floors, marking of bales for sale, packaging of tobacco for sale, and sale of tobacco by auction	No clearly stated position on collaboration regarding tobacco consumption control
4. Statutory Instrument 138 of 1968-tobacco general regulations	Regulates permits to export tobacco, prescription of noxious substances, prohibition against sale of tobacco treated with certain substances, fumigation and certificate of inspection.	No clearly stated position on collaboration regarding tobacco consumption control
5. Statutory Instrument 139 of 1968. Act No. 13 of 1994 – Registration of growers	Regulates registration of growers, registration periods, duration of registration, application for registration	No clearly stated position on collaboration regarding tobacco consumption control
6. Statutory Instrument 140 of 1968. Act No. 68 of 1977 – regulations for prescribed classes of tobacco	Regulates the prescribed classes of tobacco	No clearly stated position on collaboration regarding tobacco consumption control
7. Statutory Instrument 163 of 1992-public health tobacco regulations	Regulation concerned with labelling, prohibition of commercial advertising on billboards and newspapers, reference to health in advertising, sale of tobacco to children, prohibition of smoking in places.	Targets regulation of tobacco promotion and consumption but provides no clearly stated guidelines on the role of collaboration
8. Statutory Instrument 185 of 2008. Th local government Act No. 281 – prohibition of smoking in public places regulations	Prohibits smoking in public places	Targets regulation of tobacco promotion and consumption but provides no clearly stated guidelines on the role of collaboration

**Table A2.** Document review of tobacco-related policies.

Policies	Objective of the policy	Position on collaboration in tobacco consumption control
1. 7th National Development Plan 2017–2021	To create a diversified and resilient economy for sustained growth and socioeconomic transformation driven, among others, by agriculture, tourism, manufacturing and mining.	Departs from sectoral-based planning to an integrated multisectoral development approach, but there no mention of this with regards to tobacco consumption control
2. National Cancer Control Strategic Plan 2016–2021	This strategic plan provides guidance on the interventions needed to reduce the burden of cancers in Zambia.	Recognises the role of other sectors in controlling tobacco consumption, a key risk factor for cancers in Zambia
3. National Health in all Policies Strategic Framework _2017–21	To ensure that all sectors include health and well-being as key considerations in policy development. lays a strong foundation for the implementation of the strategies	Proposes that health is integrated in other sector policies; i.e. education, gender, water and sanitation and environmental protection, community development, social services and local government. No specific mention of tobacco
4. Zambia National Health Research Priorities_2018–2021	To align the production of research evidence to the National Health Goals and Objectives as well as provide guidance to researchers, research institutions, academic institutions, policy makers, program implementers, health development partners and other partners on Zambia’s health research focus.	Does no clearly recognise tobacco as a key priority research area among all the key themes of focus. Collaboration is key area of implementing the prescribed policy areas.
5. Zambia National NCD Strategic Plan 2013–216	To raise priority accorded to the prevention and control of NCDs in the national agenda through advocacy and multi-sectoral partnership	Recognises tobacco as a major risk factor for non-communicable diseases and proposes strategic direction to address. However, collaboration in implementing tobacco consumption control policy is not emphasised.
6. Zambia National Health Strategic Plan 2017–2021	To improve the health status of people in Zambia in order to contribute to increased productivity and socio-economic development	Not clear on strategies to address tobacco consumption in Zambia
7. Vision 2030	Zambia to become a prosperous Middle-Income Nation by 2030	Not clear on strategies to address tobacco consumption in Zambia

## Appendix B

### Interview Guide 1A

1. To evaluate governance of intersectoral action response to tobacco control in Zambia.

### Target group

1. MoH dept policy and planning,
2. MoH public health,
3. MoH Social determinants and health promotion
4. Technical working group for NCDs
5. Tobacco civil society (Researchers, NGOs)

### Purpose of the research

Thank you for agreeing to do this interview. My name is … … … … … . … … . This study aims at assessing Zambia’s response to non-communicable diseases using policy and systems analysis approaches. To help do that, we would like to know your experiences, views, submissions around the current collaborations of various sectors to address tobacco in Zambia.

### Discussion ground rules

After the completion of the consent process, which explains the study in detail and gives us permission to discuss with you, you are requested to provide answers to all the questions, if uncomfortable you may skip to other questions. The interview will last an hour. Please note that we shall be recording the information for our analysis.

Have you any questions prior to the interview?

[Turn on the recorders]

I am the interviewer … … … … … … … … … …, interviewing IDI … … … … … … Date … Start time … … … … End time

**Background Information (**kindly fill in the information below)

Sex:… …Male … … … … … … … … … … … … … … … … … . Female … … … … … … … … … … … … …

Occupation and Sector … … … … … … … … … … … … … … … … … … … … … … … … … … … … … … … … … … … … … … … … … … … … …

Experience/years in executing current duty … … … … … … … … … … … … … … … … … … … … … … … … … … … … … … … … …

Age at last birthday … … … … … … … … … … … … … … … … … … … … … … … … … … … … … … … … … … … … … … … … … … … … … … … .

**Table:**

	Main question	Probe
	Please tell me about yourself?	Your job You experience What is your current role with regards to tobacco control?
	I would like to find out the role of governance in intersectoral action to control tobacco in Zambia?	**Collaboration** Describe your understanding of collaboration Describe your understanding of intersectoral action and its role in addressing tobacco control? What are some of the collaborative/intersectoral activities that you are involved in order to address tobacco control? Is there any prior history of collaboration to act on tobacco? what was the activity? who and how was it initiated? Probe for any efforts to involve other sectors in tobacco policy development. What was their role? How should the various sectors be engaged in intersectoral action against tobacco? What sorts of incentives and motivations to encourage intersectoral action across sectors? How would describe the roles and participation of various sector stakeholders in intersectoral action on tobacco? Describe some of the factors that may facilitate intersectoral collaboration to address tobacco in Zambia? Explain the factors that may hinder intersectoral collaboration to address tobacco in Zambia? Probe for political and social cultural Probe regulatory –policy and law What would you consider to be the most important factors for collaboration on tobacco control to occur? Do you have suggestions of how intersectoral collaboration may be enhanced?**Strategic vision** How supportive is the policy and legal environment for intersectoral action on tobacco in Zambia? Describe some of the challenges you have encountered in coming up with a comprehensive tobacco policy? How can they be addressed?**Leadership and coordination** What do you understand by coordination? Please explain how intersectoral coordination is conducted in tobacco control in Zambia? If not, how should it be? What role does leadership play in fostering intersectoral action? What form of leadership is most appropriate for tobacco control in the Zambian context? What kind of coordination activities are in place/needed to address tobacco control? What structures are in place to coordinate intersectoral action on tobacco? How effective do you think are those structures? What process are in place to govern and follow up on intersectoral action on tobacco control? Describe some of the factors that may facilitate the coordination of intersectoral action on tobacco control in Zambia? Explain the factors that may hinder efforts coordination action on tobacco control across sectors? Probe for political and social cultural Probe for regulatory – Policy and law Do you have any suggestion of how coordination of action to reduce tobacco across sectors can be enhanced?
**End of Interview**		

### Interview Guide 1B

(2) To evaluate governance of intersectoral action response to tobacco control in Zambia?

### Target group

1. Local government
2. Ministry of Agriculture,
3. Ministry of Commerce,
4. Ministry of Trade,
5. Ministry of Finance,
6. Private sector (British American Tobacco, Tobacco Association of Zambia)

### Purpose of the research

Thank you for agreeing to do this interview. My name is … … … … … . … … . This study aims at assessing Zambia’s response to non-communicable diseases using policy and systems analysis approaches. To help do that, we would like to know your experiences, views, submissions around the current collaborations of various sectors to address non-communicable diseases in Zambia.

### Discussion ground rules

After the completion of the consent process, which explains the study in detail and gives us permission to discuss with you, you are requested to provide answers to all the questions, if uncomfortable you may skip to other questions. The interview will last an hour. Please note that we shall be recording the information for our analysis.

Have you any questions prior to the interview?

[Turn on the recorders]

I am the interviewer … … … … … … … … … …, interviewing IDI … … … … … … Date … Start time … … … … End time

**Background Information (**kindly fill in the information below)

Sex:… …Male … … … … … … … … … … … … … … … … … . Female … … … … … … … … … … … … …

Occupation and Sector … … … … … … … … … … … … … … … … … … … … … … … … … … … … … … … … … … … … … … … … … … … … …

Experience/years in executing current duty … … … … … … … … … … … … … … … … … … … … … … … … … … … … … … … … …

Age at last birthday … … … … … … … … … … … … … … … … … … … … … … … … … … … … … … … … … … … … … … … … … … … … … … … .

**Table:**

Main question	Probe
● Please tell me about yourself?	Your job You experience What is your current role with regards to tobacco control?
● I would like to find out the role of governance in intersectoral action to control tobacco in Zambia?	**Collaboration** Describe your understanding of collaboration Describe your understanding of intersectoral action and its role in addressing tobacco control? What are some of the collaborative/intersectoral activities that you are involved in order to address tobacco control? Is there any prior history of collaboration to act on tobacco with other sectors – MoH? what was the activity? who and how was it initiated? Probe for any efforts to involve other sectors in tobacco policy development. How should the various sectors be engaged in intersectoral action against tobacco? What sorts of incentives and motivations to encourage multisectoral action across sectors? How would describe the roles and participation of various sector stakeholders in intersectoral action on tobacco? Do you have any activities within your sector addressing tobacco – probe incorporation into policy or program Describe some of the factors that/may facilitate intersectoral collaboration to address tobacco in Zambia? Explain the factors that/may hinder intersectoral collaboration to address tobacco in Zambia? Probe for political and social cultural Probe regulatory –policy and law What would you consider to be the most important factors for collaboration and action on tobacco control to occur? Do you have suggestions of how collaboration may be enhanced?**Strategic vision** How supportive is the policy and legal environment for intersectoral action on tobacco in Zambia? Describe some of the challenges you have encountered in coming up with a comprehensive tobacco policy? How can they be addressed?
	**Leadership and coordination** What sought of intersectoral action is/should be prioritised for your sector to reduce tobacco? What are some of the capacities required for your sector to be able to implement this action? What role does leadership play in fostering intersectoral action? What form of leadership is most appropriate for tobacco control in your sector? What do you understand by coordination? Please explain how intersectoral coordination is conducted in tobacco control in Zambia? If not, how should it be? What are some of coordination activities to address tobacco control? What structure is in place to coordinate intersectoral action on tobacco? How effective is the structures and mechanisms? Are there any policies in place to facilitate the coordination intersectoral coordination? What process are in place to govern and follow up on intersectoral action on tobacco control? Describe some of the factors that/may facilitate the coordination of intersectoral action on tobacco control in Zambia? Explain the factors that/may hinder efforts coordination action on tobacco control across sectors? Probe for political and social cultural Probe for regulatory – Policy and law Do you have any suggestion of how coordination of action to reduce tobacco across sectors can be enhanced?
**End of Interview**	
